# The Mediating Roles of Sleep Quality and Physical Activity in the Association Between Smartphone Addiction and Physical Fitness Among College Students

**DOI:** 10.3390/healthcare14152433

**Published:** 2026-08-06

**Authors:** Sichao Chen, Yubo Liu, Weibing Ye, Ankang Wu, Jianwei Wu

**Affiliations:** 1 College of Physical Education and Health Sciences, Zhejiang Normal University, Jinhua 321004, China; csc0615@zjnu.edu.cn (S.C.); liuyubo0124@outlook.com (Y.L.); ywbls@zjnu.cn (W.Y.); wuankang@zjnu.edu.cn (A.W.); 2 University Hospital, Zhejiang Normal University, Jinhua 321004, China

**Keywords:** smartphone addiction, physical fitness, sleep quality, physical activity

## Abstract

**Background:** In recent years, the decline in physical fitness among college students has emerged as a public health concern, with smartphone addiction considered a potential contributing factor. However, previous studies have predominantly relied on self-reported health metrics, and potential pathways underlying its association on objective physical fitness remain to be further elucidated. Based on objective physical fitness test data, this study aimed to examine indirect associations through sleep quality and physical activity in the association between smartphone addiction and physical fitness among college students. **Method:** This cross-sectional study included 3400 undergraduates from one university in eastern China. Smartphone addiction, sleep quality, and physical activity were assessed using the Mobile Phone Addiction Index, Pittsburgh Sleep Quality Index, and International Physical Activity Questionnaire-Short Form, respectively. Physical fitness scores were obtained from the National Student Physical Health Standard. A parallel mediation model was estimated using structural equation modeling, adjusting for sex and body mass index (BMI). Indirect effects were evaluated using a nonparametric percentile bootstrap procedure with 2000 resamples. **Results:** (1) Higher smartphone addiction scores were associated with lower self-reported physical activity (*r* = −0.085, *p* < 0.001) and poorer sleep quality (*r* = 0.396, *p* < 0.001). (2) Higher objective physical fitness scores were weakly associated with greater physical activity (*r* = 0.062, *p* < 0.001) and better sleep quality (*r* = −0.042, *p* < 0.05). (3) Mediation analysis revealed that the direct association of smartphone addiction on physical fitness was non-significant (*B* = −0.014, 95% CI [−0.045, 0.015]), whereas the total indirect association through sleep quality and physical activity was statistically significant but small in magnitude (*B* = −0.022, 95% CI [−0.035, −0.010]). **Conclusions:** Higher smartphone addiction scores were indirectly associated with lower objectively assessed physical fitness through poorer sleep quality and lower self-reported physical activity, whereas the direct association was not statistically significant. However, the observed effects were small, and their practical significance should be interpreted cautiously. Longitudinal studies are needed to confirm the direction and practical relevance of these associations.

## 1. Introduction

In recent years, driven by the rapid transformation of modern lifestyles and increasing mechanization, low physical activity and high sedentary behavior have become important health concerns among university students [1,2,3]. Particularly in China, large-scale national surveys indicate a significant deterioration in objective physical fitness indicators, such as cardiorespiratory endurance and muscular strength, over the past decade, accompanied by a rising prevalence of overweight and obesity [4,5,6]. As adequate physical fitness is protective against cardiovascular disease, metabolic syndrome, and other chronic conditions, identifying modifiable factors associated with declining physical fitness has become an important priority in public health and sports medicine [7,8,9,10].

Among various potential lifestyle risk factors, smartphone addiction has garnered considerable attention within the academic community [11]. It is generally characterized by impaired control over smartphone use and associated functional difficulties across psychological, physiological, and social domains [12,13]. College students may be particularly vulnerable because of high levels of smartphone access, academic pressure, and developing self-regulation skills [14]. Problematic smartphone use has been associated with depression, anxiety, musculoskeletal discomfort, and other adverse health outcomes [15,16,17]. However, its association with objectively assessed physical fitness and the potential pathways underlying this relationship remain insufficiently understood.

Sleep quality may represent a physiological recovery pathway linking smartphone addiction with physical fitness. Bedtime screen exposure may disrupt circadian rhythms through blue-light exposure and increase cognitive or emotional arousal, thereby contributing to delayed sleep onset and reduced sleep duration [18,19,20,21]. Previous studies have associated higher smartphone addiction tendencies with poorer sleep quality and a greater risk of sleep disturbance among college students [20,22]. In turn, inadequate sleep may interfere with physiological recovery, energy regulation, and physical performance [23,24,25,26]. Poor sleep quality may therefore partly account for the association between smartphone addiction and lower physical fitness.

Simultaneously, physical activity may represent another potential pathway linking smartphone addiction with physical fitness [27,28]. Excessive engagement in smartphone-based activities may reduce the time available for exercise and other movement-related behaviors [29,30]. Problematic smartphone use has been associated with greater sedentary behavior and lower participation in physical activity [11]. Because regular physical activity contributes to cardiorespiratory endurance, body composition, and muscular fitness, lower activity levels may partly explain the association between smartphone addiction and physical fitness [9,27,28]. Although closely related, physical activity and physical fitness are distinct constructs: physical activity refers to movement behavior and energy expenditure, whereas physical fitness reflects the capacity to perform physical tasks [31]. Accordingly, physical activity was examined as a potential mediator, while objectively assessed physical fitness was treated as the study outcome.

Taken together, sleep quality and physical activity were examined as parallel mediators because they represent two complementary pathways linking smartphone use with physical fitness. Sleep quality reflects a physiological recovery pathway, as problematic smartphone use is associated with sleep disturbance, while inadequate sleep may impair physical performance [32,33]. Physical activity reflects daily movement behavior, as greater smartphone use has been associated with lower physical activity [34]. Both factors are also potentially modifiable through campus-based health interventions, although they do not represent all possible explanatory mechanisms.

Although the associations among smartphone addiction, sleep quality, physical activity, and health-related outcomes have been widely investigated, less is known about how these factors are related to physical fitness assessed under standardized objective testing conditions. Previous studies have frequently relied on self-reported health indicators, which may not fully capture college students’ objectively measured physical fitness. Accordingly, the principal contribution of the present study is the use of large-scale objective physical fitness data derived from the National Student Physical Health Standard. Sleep quality and physical activity were simultaneously incorporated into a parallel mediation framework to examine two potential indirect pathways linking smartphone addiction with objectively assessed physical fitness. Drawing upon existing theories and empirical evidence, this study proposes the following hypotheses: H1: Smartphone addiction is negatively associated with objective physical fitness among college students. H2: Sleep quality accounts for an indirect association between smartphone addiction and objective physical fitness. H3: Physical activity accounts for an indirect association between smartphone addiction and objective physical fitness. H4: Sleep quality and physical activity show parallel indirect associations between smartphone addiction and objective physical fitness.

## 2. Method

### 2.1. Participants and Procedure

This cross-sectional study was conducted at Zhejiang Normal University. Undergraduate students were recruited from public physical education courses and administrative classes across several colleges within the university using a class-based cluster recruitment approach. The minimum required sample size was estimated using Epi Info 7 (version 7.2.5.0). Based on a total population base of approximately 31,000 enrolled undergraduates at the university, with parameters set at a 5% margin of error, a 95% confidence level, and a design effect of 1.0, the calculations were as follows: (1) Assuming an expected prevalence of smartphone addiction of 21.3% among Chinese college students [35], the theoretical minimum required sample size was calculated as 255; (2) Assuming an expected prevalence of sleep disorders of 25.7% [36], the theoretical minimum required sample size was calculated as 291. The undergraduate population base comprised all full-time undergraduate students, excluding postgraduates, with data obtained from the official website of Zhejiang Normal University. The expected prevalence rates of smartphone addiction and sleep disorders were derived from studies by Long et al. [35] and Li et al. [36], respectively. The 5% margin of error was determined in accordance with the study by Charan and Biswas [37].

Data were collected in two stages. Online questionnaire data were collected through the Wenjuanxing platform from September to October 2024. Participants accessed and completed the questionnaire by scanning a QR code distributed by uniformly trained investigators. During questionnaire completion, participants entered their student identification numbers solely for linkage with their physical fitness records. Before participation, the investigators explained the study objectives, confidentiality procedures, and voluntary nature of participation, and informed consent was obtained from all participants. Standardized physical fitness assessments were subsequently conducted in November 2024 as part of the university’s physical fitness testing program. The questionnaire and physical fitness datasets were linked using student identification numbers by authorized research personnel. After successful linkage, student identification numbers were removed from the analytical dataset and replaced with study-specific codes. The identifiable linkage file was stored separately from the de-identified analytical dataset, with access restricted to designated members of the research team. All subsequent analyses were conducted using the de-identified dataset. All research procedures adhered to the Declaration of Helsinki, and the study protocol was approved by the Ethics Review Committee of Zhejiang Normal University (No. ZSRT2023129).

To ensure the quality and reliability of the data, rigorous screening criteria were applied. Questionnaires were excluded if any of the following conditions were met: (1) a completion time of less than 120 s (pre-tests indicated that even proficient respondents required a minimum of 120 s, and excessively short durations typically suggest random or inattentive responses) [38]; (2) the presence of missing data or obvious patterned responses; or (3) extreme outliers in the measured variables (e.g., biologically implausible BMI values or abnormal physical activity records). Following these screening procedures, the final analytical sample comprised 3400 participants (1216 males and 2184 females) was obtained from the initially collected 3715 questionnaires, yielding an effective retention rate of 91.5%.

### 2.2. Measures

Smartphone addiction, sleep quality, and physical activity were assessed using self-report questionnaires. Physical fitness was assessed objectively, and BMI was calculated from measured height and weight.

#### 2.2.1. International Physical Activity Questionnaire-Short Form

Participants’ physical activity levels were assessed using the International Physical Activity Questionnaire-Short Form (IPAQ-SF) [39]. The IPAQ-SF consists of seven items: the first six items survey the participants’ physical activity status, while the final item inquiries about their daily sedentary time over the past seven days. The questionnaire encompasses four domains: vigorous-intensity physical activity, moderate-intensity physical activity, walking, and sitting time, aiming to reflect the participants’ physical activity levels across varying intensities during the previous week. In accordance with the IPAQ-SF scoring protocol, the Metabolic Equivalent of Task (MET) constants assigned to different types of physical activities are as follows: walking = 3.3 METs, moderate-intensity activity = 4.0 METs, and vigorous-intensity activity = 8.0 METs. This instrument has demonstrated acceptable reliability and validity across multiple countries [39]. The specific calculation method for an individual’s total weekly physical activity score is as follows:

$$Total\;PA\;\left(MET\text{-}min/week\right)=3.3\times \left(t_{walking}\times d_{walking}\right)+4.0\times \left(t_{moderate}\times d_{moderate}\right)+8.0\times t_{vigorous}\times d_{vigorous}$$

#### 2.2.2. Mobile Phone Addiction Index

The degree of smartphone addiction among college students was assessed using the Mobile Phone Addiction Index (MPAI), developed by Leung [40]. This scale comprises 17 items divided into four dimensions: Inability to Control Craving (7 items; referring to the inability to suppress the urge to use the smartphone and excessive time spent), Withdrawal (4 items; referring to negative emotions such as anxiety when unable to use the smartphone), Escape (3 items; referring to the use of the smartphone to evade real-life problems or alleviate loneliness), and Productivity Loss (3 items; referring to the decline in academic or work efficiency due to excessive smartphone use). A 5-point Likert scale is utilized for scoring, ranging from 1 (“never”) to 5 (“always”). The total score ranges from 17 to 85, with higher scores indicating a greater tendency toward smartphone addiction. In this study, the internal consistency reliability (Cronbach’s alpha) for the total scale was 0.88, while the alpha coefficients for the four subscales ranged from 0.78 to 0.93.

#### 2.2.3. Pittsburgh Sleep Quality Index

Participants’ overall sleep quality was comprehensively assessed using the Pittsburgh Sleep Quality Index (PSQI) [41]. This scale consists of 19 self-rated items that are aggregated into seven component scores: Subjective Sleep Quality, Sleep Latency, Sleep Duration, Habitual Sleep Efficiency, Sleep Disturbances, Use of Sleeping Medication, and Daytime Dysfunction. Each component is evaluated on a scale from 0 to 3. The sum of these component scores yields a global PSQI score ranging from 0 to 21, with higher scores indicating poorer overall sleep quality. Previous large-sample validation studies conducted among Chinese college students have demonstrated that the PSQI possesses good applicability and measurement validity within domestic youth populations [42]. In the present sample, the internal consistency of the seven component scores was marginal (Cronbach’s α = 0.61). This coefficient should be interpreted considering the heterogeneous sleep dimensions represented by the PSQI and the limited variability observed in some components. Nevertheless, the relatively low internal consistency indicates potential measurement error and warrants caution when interpreting associations involving the PSQI global score.

#### 2.2.4. Objective Physical Fitness Assessment

The objective physical fitness of the participants was systematically evaluated using the National Student Physical Health Standard (NSPHS) promulgated by the Ministry of Education of China [43]. In accordance with a multidimensional health model, the standard scientifically categorizes test indicators into three primary domains: body morphology, physiological function, and physical fitness. Specifically: (1) Body morphology was assessed using BMI, which was calculated from height and weight; (2) physiological function was objectively evaluated via vital capacity tests, reflecting participants’ cardiorespiratory function; (3) physical fitness assessment was designed to account for sex differences, comprising endurance capacity (assessed by a 1000-m run for males and an 800-m run for females), muscular strength (assessed by pull-ups for males and 1-min sit-ups for females), standing long jump, sprint speed (assessed by a 50-m dash), and flexibility (assessed by the sit-and-reach test). Participant height and weight were measured using an automated electronic stadiometer and scale (Hengkangjiaye, Shenzhen, China). BMI was calculated as weight in kilograms divided by height in meters squared (kg/m^2^). During the assessment process, field tests for all indicators were strictly administered following the standardized operational protocols stipulated in the NSPHS. In the final data processing stage, individual scores for each test item were weighted and summed according to the official coefficient guidelines to calculate a total physical fitness score for each participant, based upon which their overall physical fitness level was subsequently classified into corresponding grades. Detailed testing procedures are provided in Supplementary Methods S1 and Supplementary Table S1.

### 2.3. Data Processing and Statistical Analysis

All data cleaning, processing, and statistical analyses in this study were conducted within the R programming environment (version 4.4.3), primarily utilizing core statistical packages including dplyr, psych, and lavaan [44,45]. Missing values and logically implausible observations were excluded. The total IPAQ-SF score was calculated in MET-min/week and divided by 1000 before analysis, such that one unit represented 1000 MET-min/week. This rescaling facilitated the interpretation of the unstandardized path coefficients. Because a large proportion of participants selected “do not know/unsure” or provided responses that could not be interpreted consistently on the IPAQ-SF, total sitting time was excluded from the primary model as a covariate. In the initial analytical phase, descriptive statistics were first computed to ascertain the means and standard deviations of the core variables. Independent-samples *t*-tests were then employed to compare the differences in these indicators across sexes. Subsequently, Pearson correlation analyses were conducted to examine the bivariate relationships among smartphone addiction, sleep quality, physical activity levels, and physical fitness scores, thereby providing an empirical basis for subsequent structural modeling. Because smartphone addiction, sleep quality, and physical activity were assessed using self-report instruments, Harman’s single-factor test was conducted as a preliminary assessment of potential common method variance. All items corresponding to the self-reported variables included in the analytical model were entered into an unrotated exploratory factor analysis. The percentage of variance explained by the first factor was examined to determine whether a single dominant factor was present. Because Harman’s test has limited sensitivity and cannot rule out common method bias, its findings were interpreted cautiously and were considered together with the use of an objectively assessed physical fitness outcome. A parallel mediation model was estimated using the lavaan package. To evaluate the mediating roles of sleep quality and physical activity in the relationship between smartphone addiction and objective physical fitness, smartphone addiction was specified as the independent variable (X), total objective physical fitness score as the dependent variable (Y), and sleep quality scores (M1) and physical activity (M2) as parallel mediators. Additionally, the effects of sex and BMI were included as covariates in all regression equations. The statistical significance of the direct and indirect effects was evaluated using a nonparametric percentile bootstrap procedure with 2000 resamples [46]. Unstandardized coefficients (*B*) were reported for direct, indirect, and total effects, together with their bootstrap standard errors and confidence intervals. An effect was considered statistically significant (*p* < 0.05) if the 95% confidence interval derived from the 2000 resamples excluded zero [47]. Model fit was evaluated using the chi-square statistic, comparative fit index (CFI), Tucker–Lewis index (TLI), root mean square error of approximation (RMSEA) with its 90% confidence interval, and standardized root mean square residual (SRMR). The fit indices were interpreted jointly rather than according to any single rigid cutoff. As general reference values, CFI and TLI values close to or above 0.95, RMSEA values close to or below 0.06, and SRMR values close to or below 0.08 were considered indicative of good model fit, while recognizing that the appropriateness of these criteria may vary according to model characteristics and data conditions [48,49]. Additionally, an exploratory sensitivity analysis was conducted among participants who provided an interpretable positive numerical sitting-time value.

## 3. Results

### 3.1. Descriptive Statistics and Sex Differences

The final sample comprised 3400 valid college students aged 18 to 22 years, consisting of 1216 males (35.8%) and 2184 females (64.2%). The mean BMI of the participants was 21.8 ± 3.6 kg/m^2^. Descriptive statistics for the principal variables and the results of independent sample *t*-tests for sex differences are presented in Table 1. The results indicated significant differences between sexes across several core variables. Specifically, males exhibited significantly higher weekly physical activity levels (*t* = 8.24, *p* < 0.001) and BMI (*t* = 14.93, *p* < 0.001) than females. Conversely, females scored significantly higher on smartphone addiction (*t* = −4.40, *p* < 0.001) and objective physical fitness (*t* = −23.90, *p* < 0.001) compared to males. No statistically significant sex difference was observed in sleep quality scores (*t* = −0.34, *p* = 0.736).

### 3.2. Harman’s Single-Factor Test

Because smartphone addiction, sleep quality, and physical activity were assessed using self-report measures, Harman’s single-factor test was conducted as a preliminary diagnostic of potential common method variance. The unrotated exploratory factor analysis showed that the first factor accounted for 24.4% of the total variance, which was below the commonly used reference threshold of 40%. This result suggests that no single factor dominated the covariance structure of the self-reported items. However, Harman’s test cannot exclude common method bias, and the result should therefore not be interpreted as evidence that shared-method variance was absent [50].

### 3.3. Correlation Analysis of Principal Variables

Pearson correlation analyses were conducted among smartphone addiction, sleep quality, physical activity, and objective physical fitness (Table 2). Higher smartphone addiction scores were associated with poorer sleep quality (*r* = 0.396, *p* < 0.001) and lower physical activity (*r* = −0.085, *p* < 0.001), although the latter association was small. Additionally, higher objective physical fitness scores were weakly associated with greater physical activity (*r* = 0.062, *p* < 0.001) and better sleep quality (*r* = −0.042, *p* < 0.05). The bivariate correlation between smartphone addiction and objective physical fitness was not statistically significant (*r* = −0.012, *p* > 0.05). Subsequent mediation analyses were conducted to examine potential indirect associations through sleep quality and physical activity after adjustment for the covariates.

### 3.4. Mediation Analysis of Sleep Quality and Physical Activity

Prior to evaluating individual path coefficients, the overall fit of the parallel mediation model was examined. The observed fit indices were as follows: χ^2^ (1) = 0.854, *p* = 0.355; CFI = 1.000; TLI = 1.001; RMSEA = 0.000, 90% CI [0.000, 0.044]; and SRMR = 0.003. While these values meet standard goodness-of-fit benchmarks, they are interpreted with appropriate caution given that the structural model retains only one degree of freedom (df = 1).

As shown in Table 3, higher smartphone addiction scores were associated with higher PSQI global scores, indicating poorer sleep quality (*B* = 0.087, 95% CI [0.080, 0.094]), and with lower self-reported physical activity (*B* = −0.010, 95% CI [−0.015, −0.005]). Higher PSQI global scores were associated with lower objective physical fitness scores (*B* = −0.152, 95% CI [−0.287, −0.018]), whereas greater physical activity was associated with higher objective physical fitness scores (*B* = 0.882, 95% CI [0.646, 1.111]). The direct association between smartphone addiction and objective physical fitness was not statistically significant (*B* = −0.014, 95% CI [−0.045, 0.015]).

**Figure 1:**
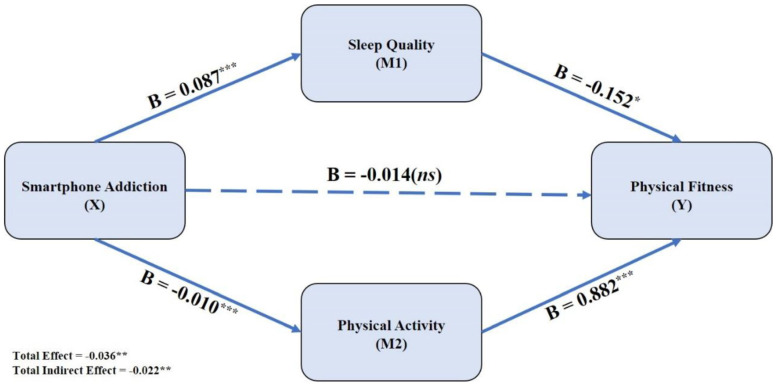
The parallel mediation model of sleep quality and physical activity in the relationship between smartphone addiction and physical fitness. NS, no significance; Sleep quality was assessed using the Pittsburgh Sleep Quality Index global score, where higher scores indicate poorer sleep quality * *p* < 0.05, ** *p* < 0.01, *** *p* < 0.001.

Analysis of the indirect effects showed significant indirect associations through both mediators. The specific indirect association through sleep quality was statistically significant (*B* = −0.013, 95% CI [−0.025, −0.002]), as was the indirect association through physical activity (*B* = −0.009, 95% CI [−0.014, −0.005]). The total indirect association was also statistically significant (*B* = −0.022, 95% CI [−0.035, −0.010]). As shown in Table 4 and Figure 1, the direct association between smartphone addiction and objective physical fitness was not statistically significant (*B* = −0.014, 95% CI [−0.045, 0.015], *p* = 0.351), whereas the total association was statistically significant (*B* = −0.036, 95% CI [−0.064, −0.009]).

### 3.5. Sensitivity Analyses

A sensitivity analysis was conducted among participants with valid self-reported sitting-time data (n = 1184). After additional adjustment for daily sitting time, the indirect effect through physical activity remained statistically significant (*B* = −0.017, 95% CI [−0.029, −0.007]), whereas the indirect effect through sleep quality was no longer statistically significant (*B* = −0.008, 95% CI [−0.028, 0.013]). The total indirect effect remained statistically significant (*B* = −0.025, 95% CI [−0.048, −0.001]), and the direct effect remained non-significant (*B* = 0.001, 95% CI [−0.047, 0.048]). Given the substantial reduction in sample size and the high proportion of participants without valid sitting-time estimates, these findings should be interpreted cautiously.

## 4. Discussion

Using standardized objective physical fitness data, this study examined whether sleep quality and self-reported physical activity statistically accounted for the association between smartphone addiction and physical fitness among college students. Significant indirect associations between higher smartphone addiction scores and lower physical fitness were observed through poorer sleep quality and lower physical activity, whereas the direct association was not statistically significant. However, the indirect associations were small in magnitude, and the correlations involving physical fitness were generally weak. Given the large sample size, statistical significance should not be interpreted as evidence of substantial practical importance. Smartphone-related behaviors are therefore likely to represent only a small part of the broader range of factors associated with college students’ physical fitness.

### 4.1. Overall Interpretation of the Findings

The findings are consistent with previous studies reporting associations between problematic smartphone use, health-related behaviors, and physical fitness outcomes [34,51]. Nevertheless, the nonsignificant bivariate correlation and direct association between smartphone addiction and physical fitness indicate that the relationship was neither strong nor directly observable in the present sample. The statistically significant total association may reflect the combined contribution of several small pathways, covariate adjustment, and unmeasured confounding rather than a substantial direct relationship. The indirect associations through sleep quality and physical activity are compatible with the possibility that lifestyle factors partly account for the relationship between smartphone addiction and physical fitness. However, the cross-sectional mediation model represents a statistical decomposition of contemporaneous associations and does not establish temporal ordering, causal mediation, or complete mediation. Alternative directions are also plausible. For example, students with poorer fitness, greater fatigue, lower motivation for exercise, or poorer sleep may engage more frequently in smartphone use. Longitudinal evidence has also suggested that problematic smartphone use and sleep disturbance may be reciprocally associated rather than linked in only one direction [32]. Other unmeasured characteristics, including psychological distress, nutritional intake, caffeine consumption, academic workload, socioeconomic conditions, and regular exercise or training habits, may also have influenced the observed associations. The participants were general undergraduate students rather than a specifically recruited athlete population, and their participation in structured training programs was not assessed. Furthermore, entering sleep quality and physical activity simultaneously as parallel mediators does not demonstrate that they are biologically independent or that they interact synergistically.

### 4.2. Sleep Quality as a Potential Indirect Pathway

The observed indirect association through sleep quality is consistent with previous evidence linking problematic smartphone use with sleep difficulties among young people [15,52]. Previous research has proposed several possible explanations, including bedtime screen exposure, delayed sleep schedules, and cognitive or emotional arousal associated with interactive smartphone activities [53,54,55]. Poor sleep has also been associated with reduced recovery and physical performance in previous studies [26]. Specifically, the study did not measure bedtime smartphone use, light exposure, sleep architecture, melatonin, cortisol, inflammatory markers, neuromuscular function, or physiological recovery. The findings therefore do not demonstrate that neuroendocrine, metabolic, or neurophysiological processes accounted for the observed indirect association. In addition, the correlation between sleep quality and physical fitness was very small, and the PSQI showed marginal internal consistency in this sample. Measurement error in the self-reported PSQI global score may therefore have affected the magnitude and precision of the sleep-related estimates. The sleep-related indirect association should also be interpreted cautiously because it was no longer statistically significant in the exploratory sensitivity analysis that additionally adjusted for sitting time among participants with valid sitting-time data. Although this analysis was based on a smaller complete-case sample, it suggests that the sleep pathway may be less robust than indicated by the primary model. Future longitudinal studies should combine device-recorded smartphone use with actigraphy or polysomnography and, where appropriate, relevant physiological measurements to clarify the temporal and biological relationships among smartphone use, sleep, and physical fitness.

### 4.3. Physical Activity as a Potential Indirect Pathway

The indirect association through physical activity is consistent with observational evidence linking greater smartphone use with lower physical activity or poorer fitness-related outcomes among college students [34]. Physical activity and physical fitness are related but distinct constructs: physical activity represents movement behavior, whereas physical fitness reflects functional capacity resulting from multiple behavioral, biological, and environmental influences [31,56]. The present findings therefore suggest that self-reported physical activity may statistically account for a small part of the association between smartphone addiction and objectively assessed physical fitness. This interpretation nevertheless requires caution. Smartphone use is not necessarily sedentary, and sedentary behavior should not be treated as equivalent to physical inactivity [57]. Although smartphone use may coincide with prolonged sitting in some contexts, the present study did not objectively determine whether smartphone use replaced physical activity or increased sedentary time. The sensitivity analysis additionally adjusting for sitting time retained the indirect association through physical activity, but it was based on a smaller subsample and should be considered exploratory. Physical activity was also assessed using the IPAQ-SF, which relies on participants’ recall of activity frequency, duration, and intensity during the preceding seven days. Recall error, social-desirability bias, and difficulty distinguishing activity-intensity categories may lead to overestimation or underestimation of actual activity. A systematic review found that IPAQ-SF estimates showed limited agreement with objective measures, indicating that its results require cautious interpretation [58]. Measurement error may therefore have influenced both the association between physical activity and fitness and the corresponding indirect estimate. Overall, the results provide preliminary evidence of small indirect associations through sleep quality and physical activity rather than confirmation of specific causal mechanisms. Campus health programs may reasonably consider problematic smartphone use, sleep habits, and physical activity together, but the present findings do not demonstrate that reducing smartphone use or modifying either mediator would necessarily improve physical fitness. Longitudinal and experimental studies using objective measures of smartphone use, sleep, physical activity, and sedentary behavior are required before stronger intervention conclusions can be drawn.

## 5. Limitations

Although this study employed a rigorous sampling design and standardized statistical methods, several limitations should be considered. First, the present study employed a cross-sectional design. Although the relationships among variables and the construction of the mediation model were grounded in established theories and prior literature, this design intrinsically serves to reveal statistical associations and potential indirect associations rather than establish strict causal inferences or temporal precedence. Second, smartphone addiction, sleep quality, and physical activity were assessed using self-report questionnaires and may therefore be affected by recall bias, social-desirability bias, reporting error, and common method variance. In particular, the IPAQ-SF requires participants to recall the frequency, duration, and intensity of physical activity during the previous seven days, which may lead to overestimation or underestimation of actual activity levels. Although physical fitness was assessed objectively, reducing same-source bias for the outcome, this does not eliminate measurement error among the self-reported variables. In addition, the PSQI demonstrated marginal internal consistency in the present sample (Cronbach’s α = 0.61). Because the PSQI global score integrates several heterogeneous sleep dimensions and some components showed limited variability, measurement error may have affected the magnitude and precision of the sleep-related associations. The estimated indirect association through sleep quality should therefore be interpreted cautiously. Future studies should combine validated questionnaires with objective measures, such as accelerometry for physical activity and actigraphy or polysomnography for sleep. Third, physical fitness is a complex outcome influenced by multiple biological, behavioral, psychological, and socioeconomic factors. Several potentially relevant confounding variables, including nutritional intake, socioeconomic status, mental health, smoking, alcohol consumption, academic workload, and genetic predisposition, were not assessed or controlled in the present model. Residual confounding may therefore have influenced the observed associations and effect estimates. Future studies should collect and adjust for a broader range of behavioral, psychological, and socioeconomic factors. Finally, participants were young undergraduate students recruited from a single public university in eastern China, which limits the generalizability of the findings. The results may not be applicable to students from other types of institutions, non-student populations, older age groups, or individuals from different regional and socioeconomic backgrounds. Future multicenter studies involving more diverse populations are needed to assess the external validity of these findings.

## 6. Conclusions

In this cross-sectional study, higher smartphone addiction scores were indirectly associated with lower objectively assessed physical fitness through poorer sleep quality and lower physical activity, whereas the direct association was not statistically significant. Although statistically significant, the indirect association was modest in magnitude, and its practical significance should be interpreted cautiously. Longitudinal studies are needed to confirm the direction and causal nature of these associations.

## Figures and Tables

**Table 1:** Descriptive statistics and independent-samples *t*-test results for sex differences in smartphone addiction, sleep quality, physical activity, physical fitness, and BMI (n = 3400).

Variables	Total (N = 3400) Mean ± SD	Male (n = 1216) Mean ± SD	Female (n = 2184) Mean ± SD	*t* Value	*p* Value	Cohen’s *d*
Smartphone Addiction (score)	36.9 ± 10.2	35.9 ± 10.2	37.5 ± 10.2	−4.40	<0.001	−0.16
Sleep Quality (PSQI global score)	3.7 ± 2.3	3.7 ± 2.2	3.8 ± 2.3	−0.34	0.736	−0.01
Physical Activity (MET-min/week)	1199.7 ± 1359.7	1454.7 ± 1511.5	1057.7 ± 1245.4	8.24	<0.001	0.30
Physical Fitness (score)	69.8 ± 9.8	64.8 ± 10.4	72.6 ± 8.3	−23.90	<0.001	−0.86
Body Mass Index (kg/m^2^)	21.8 ± 3.6	23.0 ± 4.2	21.1 ± 3.1	14.93	<0.001	0.53

Values are presented as mean ± standard deviation. Differences between male and female participants were examined using independent-samples *t*-tests. Higher PSQI global scores indicate poorer sleep quality. SD, standard deviation; BMI, body mass index.

**Table 2:** Pearson correlations analysis.

Variables	Smartphone Addiction	Sleep Quality	Physical Activity	Physical Fitness
Smartphone Addiction	1			
Sleep Quality	0.396 ***	1		
Physical Activity	−0.085 ***	−0.045 **	1	
Physical Fitness	−0.012	−0.042 *	0.062 ***	1

* *p* < 0.05, ** *p* < 0.01, *** *p* < 0.001.

**Table 3:** Path regression analysis.

Dependent Variable	Predictor	*B*	SE	*Z* Value	*p* Value	95% CI
Sleep Quality (M1)	Smartphone Addiction (X)	0.087 ***	0.004	24.91	<0.001	[0.080, 0.094]
Physical Activity (M2)	Smartphone Addiction (X)	−0.010 ***	0.002	−4.25	<0.001	[−0.015, −0.005]
Physical Fitness (Y)	Smartphone Addiction (X)	−0.014	0.015	−0.93	0.351	[−0.045, 0.015]
	Sleep Quality (M1)	−0.152 *	0.069	−2.22	0.027	[−0.287, −0.018]
	Physical Activity (M2)	0.882 ***	0.120	7.37	<0.001	[0.646, 1.111]

* *p* < 0.05, *** *p* < 0.001.

**Table 4:** Bootstrap parallel mediation analysis.

Effect Type	Path	*B*	BootSE	Bootstrap 95% CI
Direct Effect	X → Y	−0.014	0.015	[−0.045, 0.015]
Indirect Effect 1	X → M1 → Y (IE1)	−0.013 *	0.006	[−0.025, −0.002]
Indirect Effect 2	X → M2 → Y (IE2)	−0.009 ***	0.002	[−0.014, −0.005]
Total Indirect Effect	IE1 + IE2	−0.022 **	0.006	[−0.035, −0.010]
Total Effect	Direct effect + Total indirect effect	−0.036 **	0.014	[−0.064, −0.009]

* *p* < 0.05, ** *p* < 0.01, *** *p* < 0.001.

## Data Availability

The datasets generated and/or analyzed used in this study are available from the corresponding authors as the dataset contains sensitive information concerning students’ health, behavior, and physical fitness.

## Supplementary Materials

The following supporting information can be downloaded at https://www.mdpi.com/article/10.3390/healthcare14152433/s1: Methods S1 Detailed protocols for objective physical fitness assessments: S1.1. General Testing Procedures; S1.2. Height, Weight, and Body Mass Index; S1.3. Vital Capacity; S1.4. 50-m Sprint; S1.5. Sit-and-Reach; S1.6. Standing Long Jump; S1.7. Pull-Ups for Male Students; S1.8. One-Minute Sit-Ups for Female Students; S1.9. 800-m and 1000-m Endurance Runs; S1.10. Scoring and Construction of the Total Physical Fitness Score. Table S1: Summary of the administration and scoring of the physical fitness assessments.
