# Supplementary material for: The Mediating Roles of Sleep Quality and Physical Activity in the Association Between Smartphone Addiction and Physical Fitness Among College Students
Source: Healthcare (Basel). 2026 Aug 6;14(15):2433. doi: 10.3390/healthcare14152433 (PMC13464903; doi:10.3390/healthcare14152433)
Supplement: Supplementary file 1 [file healthcare-14-02433-s001.zip › healthcare-4430755-supplementary.pdf]

Supplementary Methods S1. Detailed Protocols for Objective Physical Fitness Assessments

S1.1. General Testing Procedures

Testing was organized in accordance with the National Student Physical Health Standard (NSPHS; 2014 revised edition) and the university's standardized testing instructions. Indoor assessments comprised height and weight, vital capacity, sit-and-reach, standing long jump, and the 1-min sit-up test for female students. Outdoor assessments comprised the 50-m sprint, pull-ups for male students, and the 800-m run for female students or 1000-m run for male students. Trained personnel provided standardized instructions and supervised all assessments. Participants were required to verify their identity, wear appropriate sports clothing and footwear, and refrain from testing when illness, injury, or other conditions made exercise unsafe. An adequate warm-up was completed before the running and muscular-performance tests.

S1.2. Height, Weight, and Body Mass Index

Height and weight were measured once using a calibrated electronic height-and-weight measurement system (Hengkangjiaye, Shenzhen, China). Participants removed their shoes and stood upright on the platform with their heels together, legs extended, arms relaxed at their sides, and head positioned in the Frankfurt horizontal plane. Height was recorded to the nearest 0.1 cm. Weight was measured while participants wore light clothing and stood still in the center of the platform and was recorded to the nearest 0.1 kg. Body mass index (BMI) was calculated as body mass in kilograms divided by height in meters squared (kg/m^2).

S1.3. Vital Capacity

Vital capacity was assessed using an electronic spirometer and a clean disposable mouthpiece. Participants stood upright, inhaled as deeply as possible, sealed their lips around the mouthpiece, and then exhaled continuously and completely without interruption or air leakage. Two trials were performed, and the larger value was retained. Vital capacity was recorded in milliliters.

S1.4. 50-m Sprint

Vital capacity was assessed using an electronic spirometer and a clean disposable mouthpiece. Participants stood upright, inhaled as deeply as possible, sealed their lips around the mouthpiece, and then exhaled continuously and completely without interruption or air leakage. Two trials were performed, and the larger value was retained. Vital capacity was recorded in milliliters.

S1.5. Sit-and-Reach

Participants sat with both legs fully extended, knees straight, and feet placed flat against the vertical board of the sit-and-reach apparatus. With both hands aligned, palms facing downward, and fingers extended, participants slowly pushed the measuring slider forward as far as possible without bouncing, bending the knees, or

advancing one hand ahead of the other. Two trials were completed, and the better result was retained. The score was recorded to the nearest 0.1 cm; values beyond the zero point were recorded as positive and values short of the zero point as negative.

S1.6. Standing Long Jump

Participants stood with both feet naturally separated behind the take-off line. Using an arm swing, they jumped forward from a simultaneous two-foot take-off and landed on both feet. A run-up, preparatory hop, stepping on or beyond the take-off line, or an invalid landing resulted in an invalid trial. The distance from the take-off line to the nearest point of contact on landing was recorded in centimeters. Two valid trials were allowed, and the longer distance was retained.

S1.7. Pull-Ups for Male Students

Male participants grasped a horizontal bar with a pronated grip and the hands slightly wider than shoulder width. The test began from a stationary straight-arm hang. A repetition was counted when the participant raised the body until the chin passed above the upper edge of the bar and then returned to full elbow extension. Kicking, swinging, excessive trunk movement, incomplete chin elevation, or incomplete elbow extension invalidated the repetition. The test ended when the participant could no longer complete a valid repetition or when the interval between repetitions exceeded 10 s. The total number of valid repetitions was recorded.

S1.8. One-Minute Sit-Ups for Female Students

Female participants lay supine on a mat with the knees flexed to approximately 90°, the feet secured, and the hands placed beside or behind the head according to the standardized instructions. At the start signal, participants raised the trunk until both elbows touched or passed the knees and then returned until both shoulder blades contacted the mat. The number of valid repetitions completed within 60 s was recorded. Repetitions involving incomplete trunk elevation, failure of the shoulder blades to contact the mat, or assistance from the arms were not counted.

S1.9. 800-m and 1000-m Endurance Runs

Female participants completed an 800-m run and male participants completed a 1000-m run on a measured athletics track. Participants used a standing start and began after the official signal. They were instructed to complete the prescribed distance as quickly as possible while remaining within the designated course. Timing stopped when the participant’s torso crossed the finish line. One valid trial was recorded in minutes and seconds. Participants were instructed to continue walking briefly after finishing rather than stopping abruptly.

S1.10. Scoring and Construction of the Total Physical Fitness Score

Raw results were converted to sex- and grade-specific item scores using the official NSPHS scoring tables. University students were classified into the first-/second-year or third-/fourth-year scoring group, as applicable. The weighted standard score was

calculated from the following components: BMI, 15%; vital capacity, 15%; 50-m sprint, 20%; sit-and-reach, 10%; standing long jump, 10%; pull-ups for male students or 1-min sit-ups for female students, 10%; and the 1000-m run for male students or 800-m run for female students, 20%.

Standard score = 0.15(BMI score) + 0.15(vital capacity score) + 0.20(50-m sprint score) + 0.10(sit-and-reach score) + 0.10(standing long jump score) + 0.10(strength score) + 0.20(endurance-run score).

For university students, additional points were available for performances exceeding the 100-point standard in the strength and endurance items. Male students could receive additional points for pull-ups and the 1000-m run, whereas female students could receive additional points for 1-min sit-ups and the 800-m run. Each item contributed up to 10 additional points. The total physical fitness score used in the present analysis was the sum of the weighted standard score and any applicable additional points; therefore, the theoretical maximum was 120 points. Higher scores indicated better overall physical fitness.

Supplementary Table S1. Summary of the Administration and Scoring of the Physical Fitness Assessments

**Assessment**	**Trials and retained score**	**Recorded unit**	**NSPHS weight**	**Primary validity criteria**
Height/weight and BMI	One measurement	0.1 cm; 0.1 kg; kg/m²	15% (BMI)	Barefoot; upright standardized posture; remain still
Vital capacity	Two trials; best value	mL	15%	Complete continuous expiration; no air leakage or interruption
50-m sprint	One valid trial	s (0.1 s)	20%	Standing start; start after signal; run through finish
Sit-and-reach	Two trials; best value	cm (0.1 cm)	10%	Knees extended; smooth bilateral reach; no bouncing
Standing long jump	Two valid trials; best value	cm	10%	Two-foot take-off; no run-up, hop, or line violation
Pull-ups (male)	One test; valid repetitions	repetitions	10%	Chin above bar; full elbow extension; no swinging or kicking
1-min sit-ups (female)	One 60-s test; valid repetitions	repetitions	10%	Elbows reach/pass knees; shoulder blades return to mat
1000-m run (male)/800-m run (female)	One valid trial	min:s	20%	Standing start; complete prescribed distance and course

Abbreviations: BMI, body mass index; NSPHS, National Student Physical Health Standard.
